# Medical Ethics

**Published:** 1843-04-15

**Authors:** 


### THE MEDICAL EXAMINER.

PHILADELPHIA, APRIL 15, 1843.

### MEDICAL ETHICS.

We are pleased to learn that the College of Physicians and Philadelphia Medical Society, two of the oldest and most efficient medical institutions in the country, are each engaged in framing a code of laws to regulate the professional conduct of their members. There has been, for many years past, a tacit understanding amongst the great body of physicians in Philadelphia, in reference to the general principles which should regulate their intercourse with each other. The most prominent members of the profession, for the past half century, have been men distinguished alike for their talents and attainments, and for their high sense of professional honour and integrity. Of this class were Rush, Kuhn, Wistar, Parke, Griffiths, Moore, James, Physick, Parrish, and others, who were among the founders and early members of the College of Physicians. These men gave a tone to the medical character of Philadelphia, and created by their example alone a high standard of professional morals. Under these circumstances the necessity of a written law was scarcely felt.

Within a few years past, however, the number of physicians has rapidly increased, and, as a consequence, there is a more lively competition, and stronger temptations exist to swerve from the path of rectitude and integrity. There is less community of feeling and interest, the bonds of fellowship are weakened, and old landmarks are lost sight of in the eager pursuit after fame or fortune. This state of feeling is constantly becoming more evident; and although a strong counteracting influence exists amongst a large portion of the profession, there is still an urgent necessity for a written code of laws which shall distinctly define the duties of medical men; and an infraction of which should render a physician liable to a forfeiture of any privileges or influence which he may derive from fellowship with the more honourable portion of his compeers.

*Professional* quackery is, unfortunately, a growing evil, and we cannot but hail the present movement on the part of the medical bodies mentioned, as a most timely and salutary measure, well calculated to elevate the character of the profession, and greatly to enlarge its usefulness.

We subjoin an abstract of the "rules of professional conduct" passed at the meeting of the Medical Society, held on the 1st inst. These rules were reported by a committee appointed to revise the by-laws, and are intended as a part of these laws.

The mode of procedure against such members of the Society as shall violate these provisions, is still under discussion, and will probably be decided at the meeting to be held this evening We hope the profession will be fully represented on the occasion.

"Any member of the Society shall forfeit his membership by any one of the following acts:

"1. Publishing his qualifications, trading in, holding a right in, or advertising, secret or patent medicines or instruments.

"2. Reporting his practice, including surgical operations, in other than medical works.

"3. Publishing in other than medical works, any article reflecting on the profession, or tending directly or indirectly to enhance his own merits, or undervalue those of other practitioners.

"4. Practising or sanctioning any system of quackery or imposture, including what is called Homœopathia."

---

*Death from bursting of a varix in the thigh of a pregnant woman.*—An example of this kind is recorded by Dr. Killer in the Medicin. Zeit. vom Preuss. Verein, Nov. 30, 1842.

